# Use of Antibiotics and Risk of Cancer: A Systematic Review and Meta-Analysis of Observational Studies

**DOI:** 10.3390/cancers11081174

**Published:** 2019-08-14

**Authors:** Fausto Petrelli, Michele Ghidini, Antonio Ghidini, Gianluca Perego, Mary Cabiddu, Shelize Khakoo, Emanuela Oggionni, Chiara Abeni, Jens Claus Hahne, Gianluca Tomasello, Alberto Zaniboni

**Affiliations:** 1 Oncology Unit, Oncology Department, ASST Bergamo Ovest, 24047 Treviglio (BG), Italy; 2 Medical Oncology Unit, Fondazione IRCCS Ca’ Granda Ospedale Maggiore Policlinico, 20122 Milan, Italy; 3 Medical Oncology Unit, Casa di Cura Igea, 20129 Milan, Italy; 4 Pharmacy Unit, School of Hospital Pharmacy—University of Milan, ASST Bergamo Ovest, 24047 Treviglio (BG), Italy; 5 Department of Medicine, Royal Marsden Hospital, London and Surrey, Sutton SM2 5PT, UK; 6 Pharmacy Unit, ASST Bergamo Ovest, 24047 Treviglio (BG), Italy; 7 Oncology Unit, Fondazione Poliambulanza, 25124 Brescia, Italy; 8 Division of Molecular Pathology, The Institute of Cancer Research, Sutton, London SM2 5NG, UK; 9 Niguarda Cancer Center, Grande Ospedale Metropolitano Niguarda, 20162 Milan, Italy

**Keywords:** cancer, antibiotics, meta-analysis, risk factor

## Abstract

The association between antibiotic use and risk of cancer development is unclear, and clinical trials are lacking. We performed a systematic review and meta-analysis of observational studies to assess the association between antibiotic use and risk of cancer. PubMed, the Cochrane Library and EMBASE were searched from inception to 24 February 2019 for studies reporting antibiotic use and subsequent risk of cancer. We included observational studies of adult subjects with previous exposure to antibiotics and available information on incident cancer diagnoses. For each of the eligible studies, data were collected by three reviewers. Risk of cancer was pooled to provide an adjusted odds ratio (OR) with a 95% confidence interval (CI). The primary outcome was the risk of developing cancer in ever versus non-antibiotic users. Cancer risk’s association with antibiotic intake was evaluated among 7,947,270 participants (*n* = 25 studies). Overall, antibiotic use was an independent risk factor for cancer occurrence (OR 1.18, 95%CI 1.12–1.24, *p* < 0.001). The risk was especially increased for lung cancer (OR 1.29, 95%CI 1.03–1.61, *p* = 0.02), lymphomas (OR 1.31, 95%CI 1.13–1.51, *p* < 0.001), pancreatic cancer (OR 1.28, 95%CI 1.04–1.57, *p* = 0.019), renal cell carcinoma (OR 1.28, 95%CI 1.1–1.5, *p* = 0.001), and multiple myeloma (OR 1.36, 95%CI 1.18–1.56, *p* < 0.001). There is moderate evidence that excessive or prolonged use of antibiotics during a person’s life is associated with slight increased risk of various cancers. The message is potentially important for public health policies because minimizing improper antibiotic use within a program of antibiotic stewardship could also reduce cancer incidence.

## 1. Introduction

Antibiotics are antimicrobial substances that are active against bacteria and represent the most important armamentarium for fighting bacterial infections. In general, management of patients with suspected bacterial infections consists of initiation of empiric therapy (i.e., before the availability of definitive culture and sensitivity data), followed by adjustment once microbiology information becomes available. In particular, isolation of bacteria from clinical samples yields information that can be used to guide the selection of appropriate regimens based on prior knowledge of bacterial susceptibility to certain antibiotics. Recent advances in knowledge have provided information that antibiotics can influence an individual’s health status via the concomitant damage of bacteria that usually live in healthy humans, the microbiota. These organisms and their genes, metabolites, and interactions with one another, as well as with their host collectively, represent our microbiome [1]. Despite the established usefulness of antibiotics in healthcare, variations in gut microbiota have been implicated in the pathogenesis of systemic diseases. Dysbiosis of gut microbiota is associated not only with intestinal disorders but also with numerous extra-intestinal diseases such as metabolic and neurological disorders [2], particularly when antibiotics are taken during the early years of life. Alteration in microbiome composition depends on the antibiotic class, dose, duration of exposure, pharmacological action, and target bacteria.

Neoplastic conditions could be affected or driven by disturbances in gut microbiota. For example, structural fecal bacterial distinction between colorectal cancer (CRC) patients and healthy volunteers have been demonstrated [3]. Genomic analysis identified an association between *Fusobacterium* spp. and colorectal cancer [4]. Indeed, *Fusobacterium* spp. may contribute to tumorigenesis by an inflammatory-mediated mechanism, but the precise role of *Fusobacteria* in colorectal carcinoma pathogenesis requires further investigation. All these findings suggest that alterations in CRC microbiota may contribute to the etiology of colorectal cancer [4]. Antibiotic exposure, even for short periods and especially during infancy, has long-lasting effects on the microbiota, which may predispose the host to a variety of chronic diseases including cancer [5,6]. This risk must be better understood as it could potentially be of paramount importance to public health strategies.

Given the enormous interest and implications for the community, we aimed to evaluate whether antibiotic use represents an independent risk factor for the development of solid tumors and lymphomas in adult humans through a systematic review and meta-analysis of epidemiological studies.

## 2. Materials and Methods

### 2.1. Study Selection and Inclusion Criteria

We followed the Meta-Analysis of Observational Studies in Epidemiology (MOOSE) reporting guidelines [7]. We performed a systematic literature search in MEDLINE, the Cochrane Library and EMBASE from inception to 24 February 2019, without language restriction, for observational studies of adults with previous exposure to antibiotics and incident cancer diagnoses and papers describing cases of incident leukemias only. We excluded studies that included participants with any prior history of malignancy or those with known concomitant antibiotic use at the time when cancer was diagnosed. We conducted the search using broadly defined medical subject headings: (Carcinoma OR neoplasms OR sarcoma OR melanoma OR lymphoma) AND risk AND antibiotics. We searched bibliographies of key articles in the field. Three authors (FP, MG and AG) independently screened the abstracts of the search results and independently assessed the remaining full-text articles for eligibility. Any disagreement was resolved with the help of a senior author (AZ).

### 2.2. Data Extraction

For each of the eligible studies, the following data were collected: Author name, year of publication, country, number of patients (including cases and controls for case–control studies), type of study, type of analysis, exposure (cumulative time on treatment and/or number of prescribed doses), type of antibiotics, type of incident cancer evaluated and covariates for odds ratios (ORs) adjustment. In the case of studies with potentially overlapping populations, the largest or most up-to-date study was included. Risk of bias in individual studies was assessed independently by four local reviewers (FP, MG, AG and GP) and by an external reviewer (JCH) with the Newcastle-Ottawa Scale for retrospective studies [8]. We rated studies as having low risk of bias if they had adjustment for age, sex and and/or tobacco use, provided detail on exposure assignment (for duration of antibiotic use or number of prescriptions), and defined type of antibiotics and/or type of cancer associated with risk. The GRADE (Grading of Recommendations, Assessment, Development and Evaluation) approach was used to assess the strength of evidence. The level of evidence was graded as high, moderate, low or very low. Observational studies received an initial grade of low. Three pre-specified criteria upgraded the certainty of the evidence: When a large magnitude of effect was present, when there was a dose-or time–response effect of exposure or when effect size (OR) was adjusted for potential confounders (e.g., age, sex and/or smoking history).

### 2.3. Statistical Analysis

Three alternative analyses were designed: (i) Ever-use versus never-use meta-analysis; (ii) latency period analysis—that is, the time elapsed between last antibiotic use and incident cancer diagnosis (the highest interval versus no use); and (iii) a dose–response analysis with comparison of a higher number of prescriptions versus none and longer duration of antibiotic exposure versus no antibiotics for each trial. The effect estimates (ORs) were extracted from each publication. Results were displayed in a standard forest plot. Concerning the main analyses, only the adjusted ORs were extracted from each study (case control or cohort, respectively) to minimize the effects of confounding variables. The fixed-effects model (Mantel–Haenszel method) and the random effects (DerSimonian–Laird) model were used to calculate the pooled OR [9]. Heterogeneity was evaluated through the I-squared test; in case no significant heterogeneity was detected, the fixed-effects model was chosen. Publication bias was assessed with Begg’s and Egger’s statistical tests and the respective funnel plot displayed [9]. In addition, a subgroup analysis was performed according to type of neoplasm and type of antibiotic if at least three publications provided data for each cancer and drug.

The analysis was performed with Review Manager (RevMan) version 5.3 (Copenhagen: The Nordic Cochrane Centre, the Cochrane Collaboration, 2014) and Comprehensive Meta-Analysis software version 3.3.070 (20 November 2014).

## 3. Results

**Figure 1:**
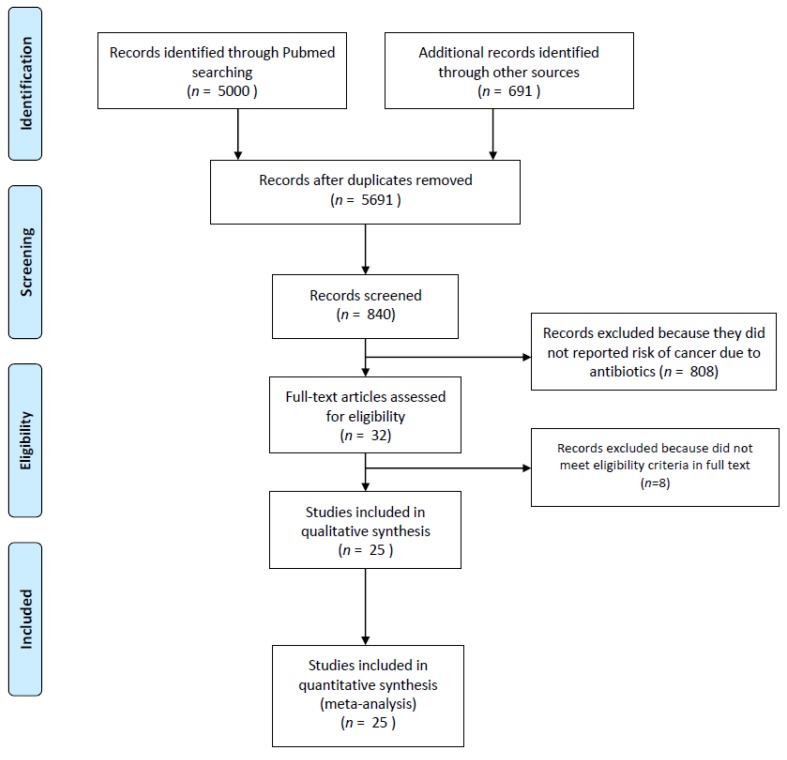
Flow diagram summarizing the literature search.

A total of 5691 records were retrieved, of which 25 observational studies (*n* = 7,947,270 patients) met eligibility criteria and were included in the meta-analysis (Figure 1; Table 1 and Table 2) [10,11,12,13,14,15,16,17,18,19,20,21,22,23,24,25,26,27,28,29,30,31,32,33,34].

Five were cohort studies, and 20 were case control studies. Overall, 368,934 cancer cases were recorded (4.6% of the total). A total of 17 incident neoplastic conditions and eight antibiotic classes (beta-lactams, cephalosporins, macrolides, tetracyclines, fluoroquinolones, nitrofurantoins, sulfonamides and nitroimidazoles) were associated with cancer incidence in at least three papers. The number of patients in each study ranged from 260 to 3,112,624 (median 18,035). The observation intervals ranged from 4.7 to 20 years. All studies except one included subjects from Western countries. Fourteen trials (56%) were of moderate to high quality according to the NOS scale.

### 3.1. Primary Analysis: Overall Cancer Incidence

**Figure 2:**
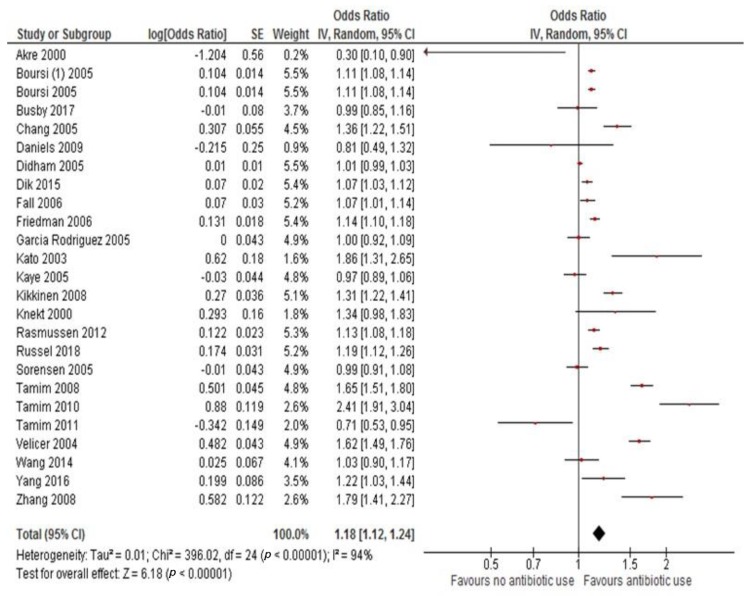
Odds ratio for cancer risk associated with antibiotic use.

All studies were used to evaluate the pooled OR for risk of cancer in ever versus non antibiotic users. The exposure to antibiotics increased the risk of cancer by 18% (adjusted OR 1.18, 95%CI 1.12–1.24, *p* < 0.001). There was evidence of high heterogeneity, therefore, a random effect model was used (Figure 2). In two papers, some cases of leukemias were included (*n* = 2725, 0.7% of the total observed cancers); after recalculating the ORs of these publications without these patients, the result was similar.

### 3.2. Secondary Analysis: Latency Period and Risk of Cancer

Eight trials evaluated the relationship between time elapsed since last antibiotic use and new incident cancer diagnoses (highest versus lowest interval). There was no association between a longer latency period and risk of cancer (adjusted OR 1.14, 95%CI 1.05–1.24; *p* = 0.001).

### 3.3. Tertiary Analysis: Correlation with Prescriptions and Risk of Exposure

Number of prescriptions (the highest number versus none or the lowest number) and duration on antibiotic therapy were most strongly associated with cancer risk (adjusted OR 1.28, 95%CI 1.14–1.44, *p* < 0.001 and adjusted OR 1.31, 95%CI 1.11–1.54, *p* = 0.001).

### 3.4. Subgroup Analysis

Table 3 reports the results of subgroup analysis. The greatest risk of cancer following antibiotic exposure was observed for lung cancer (adjusted OR 1.29, 95%CI 1.03–1.61, *p* = 0.02), lymphomas (adjusted OR 1.31, 95%CI 1.13–1.51, *p* < 0.001), pancreatic cancer (adjusted OR 1.28, 95%CI 1.04–1.57, *p* = 0.019), renal cell carcinoma (adjusted OR 1.28, 95%CI 1.1–1.5, *p* = 0.001), prostate cancer (adjusted OR 1.25, 95%CI 1.1–1.4, *p* < 0.001), and multiple myeloma (adjusted OR 1.36, 95%CI 1.18–1.56, *p* < 0.001). The antibiotic classes with the strongest association were beta lactams adjusted (OR 1.15, 95%CI 1.12–1.19, *p* < 0.001), cephalosporines (adjusted OR 1.19, 95%CI 1.13–1.25, *p* < 0.001), macrolides (adjusted OR 1.11, 95%CI 1.06–1.16, *p* < 0.001) and quinolones (adjusted OR 1.15, 95%CI 1.09–1.21, *p* < 0.001). Results were similar for cohort and case-control studies.

### 3.5. Publication Bias

**Figure 3:**
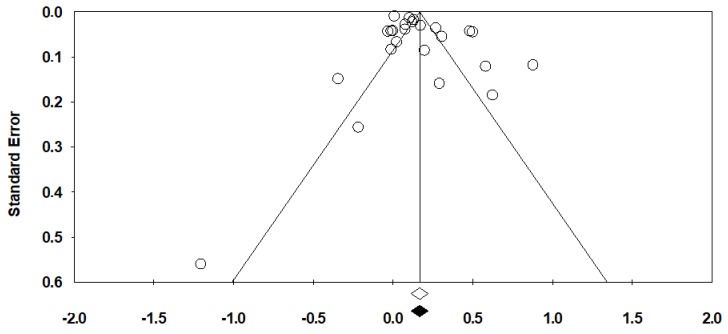
Funnel plot for publication bias for the overall risk of cancer with antibiotic use.

There was no evidence of publication bias with Begg’s test (*p* = 0.48; Figure 3). Egger’s test, however, was significant (*p* = 0.03). According to the trim and fill method, which looks for missing studies based on a random-effects model, no studies are potentially missing.

### 3.6. Strength of Evidence

There is moderate evidence that previous use of antibiotics during a person’s life is associated with a slightly increased risk of some cancers (excluding leukemia). The analysis is derived from 25 case-control or cohort studies with mainly low-moderate risk of bias in 56% of studies. There is moderate to low evidence that this risk is associated with the number of antibiotic prescriptions and duration of antibiotic exposure respectively (*n* = 21 and *n* = 6 studies with 47% and 50% associated with high risk of bias). There is moderate evidence that antibiotic use slightly increases the risk of hematological (multiple myeloma and lymphoma), gastrointestinal (colorectal, hepatobiliary, pancreatic and gastric cancers), lung and genitourinary cancers (prostate, bladder, and kidney). There is very weak evidence that the risk is increased for breast and other cancers such as gynecological cancers and melanoma. Finally, there is moderate evidence that this risk is associated with specific classes of antibiotics (macrolides, beta-lactams, quinolones, sulphonamides and cephalosporins) but low or insufficient evidence of associations with the other analyzed classes.

## 4. Discussion

In this analysis based on 7,947,270 individual participants from 25 observational studies, antibiotic use was associated with an 18% increased risk of cancer. The highest risk was found in individuals with a long duration of antibiotic exposure or in those receiving higher doses. There was a 30% increased incidence of lung, hematological, pancreatic and genitourinary cancers compared to controls due to increased antibiotic exposure. Conversely, our study found no association between esophageal or cervical cancer and antibiotic use and a small increase in risk for CRC, gastric cancer and melanoma. To our knowledge, this is the most up to date and extensive meta-analysis that assessed the association of different doses/timing of antibiotic exposure with various incident cancer diagnoses. In a published meta-analysis of five case-control studies, antibiotic use was associated with an increase in breast cancer risk (OR = 1.17, 95%CI 0.99–1.39, *p* < 0.001) but the causality of this association remained elusive [36]. Influence of the gut microflora or a direct effect of some antibiotics on mammary glands has been postulated, but data are sparse. 

Antibiotics may influence cancer risk through several mechanisms. First, as mentioned above, the gut microbiota is not a simple intestinal layer but is a key regulator of digestion along the gastrointestinal tract. Commensal bacteria also play a pivotal role in the extraction, synthesis, and absorption of many nutrients and metabolites, including bile acids, lipids, amino acids, vitamins, and short-chain fatty acids. The gut microbiota has a crucial immune function against pathogenic bacterial colonization, inhibiting their growth, consuming available nutrients and/or producing bacteriocins. Gut microbiota also prevent bacterial invasion by maintaining integrity of the intestinal epithelium. All these functions can be altered when antibiotics are consumed, and consequently, systemic inflammation can arise and latent cancer cells can grow [37]. Several microbial agents have been tested as cancer treatments in human and mouse preclinical models—in particular, those with anticancer properties (e.g., *Bacillus* of Calmette and Guerin) [35]. Antibiotics interfere with the interaction between the microbiome and the immune system, potentially resulting in reduced immune surveillance [35]. Similarly, the response to immune checkpoint inhibitors relies on the gut microbiota’s composition, and in patients treated with antibiotics during immunotherapy, clinical outcomes are consistently worse [38,39]. In summary, the microbiota can confer protection against pathogens, a phenomenon referred to as colonization resistance, which can be severely impaired by antibiotic treatments.

For some cancers, a specific association with local microbiota is described [40,41,42,43]. Mao and colleagues explored the possible links between dysbiosis and carcinogenesis and hypothesized that chronic inflammation linked to altered microbiota can be a trigger for lung cancer [41]. Similar data were published for genitourinary and pancreatobiliary cancer and for lymphomas [40,42,43,44]. A different interaction between antibiotics and colorectal or gastric cancer risk could exist. In this meta-analysis, exposure to antibiotics was associated with an 8% and 6% increase in risk for colorectal and gastric cancer, respectively. It has been shown that *Fusobacterium nucleatum* is associated with intestinal tumorigenesis, modulates the tumor-immune microenvironment and can respond to the antibiotic metronidazole by reducing cell proliferation [45]. There is also evidence that *E. coli* infection may promote chronic inflammation which leads to cell proliferation and tumor formation. The role of *H. pylori* in the pathogenesis of gastric cancer is well described. It could therefore be postulated that eradication of *H. pylori* and *Fusobacterium* or *E. coli* with prior use of antibiotics may reduce the risk of gastric and CRC respectively. However, despite these hypotheses, other risk factors probably related to a dysregulated gut or gastric microbiota can augment gastric cancer and CRC incidence in adults. In the present analysis, a protective effect is somewhat observed with cervical cancer, where risk is reduced by 25% by previous antibiotic use. Even if Human Papillomavirus (HPV) is the main factor involved in the pathogenesis of cervical cancer, *Chlamydia trachomatis* infection represents an independent risk factor, in particular when associated with HPV [46]. It is likely that the detection and treatment of this gynecological infection may reduce cervical carcinogenesis and the risk of clinically evident cancer.

According to the antibiotic classes implicated in putative risk of cancer, higher risk is associated with beta-lactams, cephalosporins and fluoroquinolones. Although the risk is quite similar, with no class associated with an excessive risk above the general pooled weighted ratio, these three classes are the most frequently investigated and used in the community, with insufficient data found for aminoglycosides and lincosamides. A broad range of antibiotics has been shown to transiently or permanently alter the composition of healthy adult microbiotas, usually via depletion of one or several species [47]. Amoxicillin exposure may cause marked changes in microbiome composition that last approximately 30 days on average and have been observed for more than two months in some subjects [48]. Massive shifts have also been reported during an oral course of ciprofloxacin, with the changes persisting for several weeks [49]. An emerging issue is the emergence of *E. coli*–related ciprofloxacin resistance [50]. It is possible that *E. coli* resistance is a facilitating factor associated with CRC [51]. Antibiotic resistance is a hot topic issued in the present years, but our data do not concern on the argument and, in particular, if any resistance may be associated with higher cancer incidence. However, since the number of prescriptions and the duration of antibiotic therapy are correlated with a larger risk, this possibility may be more than a hypothesis, and the emergence of resistant microorganism could be implicated. The studies included, however, refer to decades ago and this implies that more novel antibiotic classes, may beneficiate the excess of cancer events here described. 

Our study has significant strengths. First, to our knowledge, this is the largest meta-analysis of adult subjects exposed or not during their lifetimes to antibiotics for various conditions and evaluated for incidence of cancer (solid tumors and lymphomas). In this meta-analysis which included approximately 8 million subjects, we found an 18% increase in the risk of various cancers. This risk is notably increased (30%) in those exposed several times or for more extended periods during previous years. Second, we used multivariate-based ORs adjusted for other comorbidities, age, gender, sex, body mass index, concomitant drugs or other medical variables. We therefore defined antibiotic use as an independent risk factor for cancer.

However, our study has some limitations. First, this is not an individual patient data meta-analysis. An individual participant data analysis is not subject to the potential bias that arises in a study-level meta-analysis and would be the optimal approach to combine evidence across multiple studies and perform time-to-event analyses. Second, personal health conditions are partially unknown at the time of cancer diagnosis, and other risk factors such as diet, lifestyle, pollution or other chronic conditions that can increase baseline risk or influence the normal microbiome cannot be excluded. Third, seven studies did not report risk according to antibiotic class. Fourth, the data refer to cancer incidence only, so stage and/or pathology report and outcome are not available. Fifth, only one study included patients of Asian origin, so generalization to the worldwide population is not possible. Finally, in 12 studies, the median observation period (follow-up) was not reported.

Many antibiotics prescribed in hospitals are unnecessary or inappropriate. Some scientific societies have provided authoritative statements or guidelines to guide clinicians toward better antibiotic use. The World Health Organization defined a global action plan to ensure optimal treatment and prevention of infectious diseases with adequate and safe medicines that are quality-assured, used responsibly and accessible to all who need them (https://www.who.int/antimicrobial-resistance/global-action-plan/en/). In a similar position paper, the European Surveillance of Antimicrobial Consumption Network joined the European Union in providing guidelines on the prudent use of antimicrobials in humans (https://ecdc.europa.eu/sites/portal/files/media/en/publications/Publications/EU-guidelines-prudent-use-antimicrobials.pdf). The term “antimicrobial stewardship” refers to policies, education strategies and interventions aimed at optimizing antimicrobial use. In this way, inappropriate prescriptions can be reduced with more guidelines on the use of antibiotics in both ambulatory [52] and hospital [53] settings. In the near future, we anticipate that manipulation of the human microbiome will be combined with antineoplastic treatments to improve prognosis and likelihood of cure [54]. Fecal microbiota transplantation, prebiotic and/or probiotic formulations and other types of drug and dietary-based interventions, such as caloric restriction or fiber intake, will potentially aid the anticancer response in humans [54]. 

## 5. Conclusions

In conclusion, antibiotic use may be associated with an excess of incident cancer diagnoses and lymphomas, in particular, with overuse or prolonged exposure of main antibiotic classes (e.g., beta-lactams, cephalosporins and fluoroquinolones). These data derive from a large number of patients included in observational studies. Despite being associated with obvious bias, this information should encourage clinicians to adopt appropriate use of these drugs to treat infections according to published guidelines.

## Figures and Tables

**Table 1:** Characteristics of the included studies.

Author/Year	Type of Study	Country	N° pts	Cases	Controls	OR/RR for Risk	Type of Analysis	Adjustment Covariates	NOS	RoB
**Akre/2000** [10]	Case-control	Sweden	636	174	462	0.3 (0.1–0.7)	-	Gender, age, history of gastric resection, and regular use of aspirin	7	Mod
**Boursi/2015** [11]	Case-control	UK	103,044	20,990	82,054	1.11 (1.08–1.14)	Days of use, type of antibiotics, n° prescriptions	Diabetes mellitus, BMI, smoking history, alcohol consumption, chronic use of Aspirin/NSAIDs, and performance of screening colonoscopy.	6	Mod
**Boursi/2015** [13]	Case-control	UK	615,951	125,441	490,510	1.11 (1.08–1.14)	Time from 1st antibiotic use, type of antibiotics, n° prescriptions	Different according to cancer type (see full text)	6	Low
**Busby/2017** [14]	Case-control	Scotland	18,035	3098	14,937	0.99 (0.84–1.17)	N° prescriptions	Statin and aspirin use, and the presence of myocardial infarction, heart failure, peripheral vascular disease, cerebrovascular disease, connective tissue disease, dementia, chronic obstructive pulmonary disease, rheumatoid arthritis, diabetes, renal disease and liver disease, age, general practice and year of diagnosis	6	Mod
**Chang/2005** [15]	Case-control	Denmark and Sweden	6242	3055	3187	1.36 (1.22–1.53)	N° prescriptions	Age, sex, country	5	Mod
**Daniels/2009** [16]	Case-control	New Zealand	260	65	195	0.806 (0.487–1.33)	N° prescriptions	Age group, race, years of enrollment, and number of visits	5	Mod
**Didham/2005** [32]	Case-control	USA	12,00,000	6500	1.193.500	1.01 (0.99–1.02)	Years of use, type of antibiotics	Age	5	Mod
**Dik/2016** [17]	Case-control	Netherland	20,017	4029	15,988	1.08 (1.023–1.14)	Days of use, n° prescriptions	Age, sex, insulin-independent diabetes, insulin-dependent diabetes, and the use of proton pump inhibitors, acetylsalicylic acid, nonsteroidal anti-inflammatory drugs, blood lipid-lowering agents, estrogens, and immunosuppressive drugs	6	Low
**Fall/2006** [18]	Retrospective cohort	Sweden	501,757	645	-	1.08 (1–1.17)	Sex, age, follow up, type of infection, type of bacteria	Comorbidities	8	High
**Friedman/2006** [19]	Retrospective cohort	US	2,130,829	18521	-	1.14 (1.1–1.18)	Days of use, type of antibiotics, hormone use	Days of use, hormone use	8	High
**Garcia Rodriguez/2005** [20]	Case control	Spain	23,708	3708	20,000	1 (0.92–1.09)	Days of use, n° prescriptions, type of infection	Age, calendar year, body mass index, alcohol intake, hormone replacement therapy, use of NSAIDs, prior benign breast disease, time under observation, and utilization of healthcare services.	7	Mod
**Kato/2003** [21]	Case control	US	839	376	463	1.87 (1.3–2.7)	N° prescriptions, type of infection	Age, family history of hematologic cancer, college education, smoking status, average frequency of use of pain-relieving drugs, surrogate status and year of interview.	8	Low
**Kaye/2005** [22]	Case control	US	7559	1268	7291	0.97 (0.89–1.06)	N° prescriptions, type of antibiotics	BMI, use of hormone replacement therapy, history of benign proliferative breast disease, frequency of mammograms, and frequency of visits to the general practice	7	Mod
**Kikkinen/2008** [23]	Retrospective cohort	Finland	3,112,624	134,070	-	1.31 (1.22–1.42)	Type of cancer, n° prescriptions, years of duration, time from 1st antibiotic use	Age, sex	7	Low
**Knekt/2000** [34]	Retrospective cohort	Finland	9461	157	-	1.34 (0.98–1.83)	Age, bacteriuria, follow up	Age, region type, education, marital status, body mass index, parity, smoking, height, alcohol use and screening positive for bacteriuria.	10	Mod
**Rasmussen/2012** [24]	Retrospective cohort	Denmark	13,602	13,602	-	1.13 (1.08–1.19)	Type of antibiotics, n° prescriptions, time from 1st antibiotic	Age, sex, calendar period	9	Low
**Russel/2018** [25]	Case-control	Sweden	52,568	8762	43,806	1.19 (1.12–1.27)	Type of antibiotics, n° prescriptions, time from 1st antibiotic	Civil status, education, CCI and time between 1st antibiotic and event	6	High
**Sorensen/2005** [28]	Case-control	Denmark	30,008	2728	27,280	0.99 (0.91–1.06)	Type of antibiotics, n° prescriptions	Age at first birth, parity, and use of postmenopausal hormone replacement therapy	5	High
**Tamim/2008** [12]	Case-control	Canada	15,495	3099	12,396	1.65 (1.51–1.80)	N° prescriptions, type of antibiotic	Age, time of diagnosis and exposure to antibiotics during the other time periods	5	High
**Tamim/2010** [31]	Case-control	Canada	20,260	4052	16,208	2.41 (1.91–3.04)	N° prescriptions, type of antibiotic	Age and time of diagnosis	5	High
**Tamim/2011** [26]	Case-control	Canada	6125	1225	4900	0.71 (0.53–0.95)	N° prescriptions, type of antibiotic	Age, time of diagnosis, and antibiotic exposure in other periods	5	High
**Velicer/2004** [27]	Case-control	US	10,219	2266	7953	1.62 (1.48–1.76)	N° prescriptions, days of used, type of antibiotic	Age, level of education, race, length of enrollment, number of primary and specialty health care visits, pharmacy co-payment status, age at menarche, parity, age at first birth, body mass index, first-degree family history of breast cancer, mammographic breast density, prior hysterectomy, menopausal status, age at menopause, and use of oral contraceptives and postmenopausal hormones	5	High
**Wang/2014** [33]	Case-control	Taiwan	27,860	5572	22,288	1.02 (0.89–1.17)	N° prescriptions, type of antibiotic	Age, gender, socioeconomic status and numbers of stool occult blood tested	5	High
**Yang/2016** [35]	Case-control	UK	5835	1195	4640	1.22 (1.03–1.44)	N° prescriptions, type of antibiotic	BMI, smoking status, alcohol-related disorders, hepatitis B or C virus infection, diabetes, rare metabolic disorders, and use of anti-diabetic medications, paracetamol, and statins	5	High
**Zhang/2008** [30]	Case-control	UK	14,336	4336	10,000	1.79 (1.41–2.26)	N° prescriptions, type of antibiotic	Smoking status, smoking cessation interventions, episodes of different types of infection, history of COPD, asthma, body mass index, alcohol intake, and indicators of health care utilization	5	High

**Legend**: OR, odds ratio; RR, risk ratio; NSAID, non steroideal anti-inflammatory drug; BMI, body mass index; CCI, Charlson Comorbidity Index; pts, patients; mod: Moderate; NOS, Nottingham-Ottawa-Scale; COPD, chronic obstructive pulmonary disease; RoB, risk of bias.

**Table 2:** Characteristics of included studies with antibiotics classes, cancers analyzed and prescriptions.

Author/Year	Median Follow Up	N° of Prescriptions (Duration of Treatment)	Antibiotics Considered	Cancers Analyzed	Different Time Intervals from Last Antibiotic Use and Cancer Events (Years)
**Akre/2000** [10]	8 years	NR	NR	Gastric	NR
**Boursi/2015** [11]	6.5 years	1–5, 5–10, >10 course (1–14, 14-56, 56+ day duration)	Nitroimidazoles, penicillins, tetracyclines, macrolides, quinolones, cephalosporins, sulfonamides	Colorectal	0–1; >1
**Boursi/2015** [13]	4.7–7 years	1, 2–5, >5 courses	Penicillins, cephalosporins, macrolides, tetracyclines, sulfonamides, quinolones and nitroimidazole	Breast, Oesophagus, Gastric, HCC, Biliary, Gallbladder, Pancreas, Prostate, Renal, Bladder, Melanoma, Cervix, Osteosarcoma, MM	1–5, 5–10, >10
**Busby/2017** [14]	5.5 years	1, 2+	Tetracyclines	Gastroesophageal	NR
**Chang/2005** [15]	NR	1–2, 3–5, 6–10, 11+	NR	NHL	>2
**Daniels/2009** [16]	NR	1–25, 26–50, 51–100, 100+	Macrolides, tetracyclines, penicillins, sulfonamides, ciprofloxacin, levofloxacin (data not reported separately)	Prostate	NR
**Didham/2005** [32]	NR	NR (≥2 years)	Macrolides, tetracyclines, penicillins, cephalosporins, sulfonamides, nitrofurantoin, others	Bladder and renal, brain and central nervous system, breast, colorectal, female reproductive system, leukemia, liver, pancreas and other digestive, lung and respiratory, lymphoma (non hodgkin’s), oral cavity, pharynx, oesophagus, other, prostate, skin (melanoma), skin (neoplasms), stomach and small intestine	NR
**Dik/2016** [17]	5 years	1.2, 3–4, 5–7, ≥8	Tetracyclines, penicillins, sulfonamides, macrolides, quinolones, nitrofurantoin	Colorectal	
**Fall/2006** [18]	11.8 years	< vs. ≥3/times year	NR	Non-cardia gastric cancer	1–4, 5–9, 10–14, 15–19, 20+
**Friedman/2006** [19]	9.4 years	NR (<50, 51–100, 101–500, 501–1000, >1000 days duration)	Penicillins, Tetracyclines, Macrolides, Quinolones, Cephalosporins, Lincosamides, Aminoglycosides, Sulfonamides, Metronidazole, Isoniazid, Rifampin, Nitrofurantoin	Breast	
**Garcia Rodriguez/2005** [20]	At least 1 year	1–10, 11–25, 26+	NR	Breast	NR
**Kato/2003** [21]	2–20 years	1, 2–4, 5–8, 9–17, 18–35, 36+	NR	NHL	>2
**Kaye/2005** [22]	94 months	NR (1–50, 51–100, 101–500, 500+ days duration)	Penicillins, Tetracyclines, Macrolides, Cephalosporins	Breast	NR
**Kikkinen/2008** [23]	7 years	0–1, 1–5, ≥6 (1–3 years duration)	NR	Hematological, head & neck, gastrointestinal, thoracic, genitourinary, SNC, skin, bone, endocrine, breast, gynecological	NR
**Knekt/2000** [34]	18 years	NR	NR	Breast	NR
**Rasmussen/2012** [24]	13 years	1, 2, 3, 4, 5+	Tetracyclines, sulfonamides, penicillins, macrolides, quinolones	NHL, MM	
**Russel/2018** [25]	NR	1–3, 4–6, 7–9, 10+	Sulfonamides, cephalexin, doxycycline, nitrofurantoin, quinolones, amoxicilline/clavulanate.	Prostate	6–12 months, 1–2, 3–4, 5+
**Sorensen/2005** [28]	NR	1–5, 6–10, >10	Penicillins, tetracyclines, macrolides, quinolones, cephalosporins, sulfonamides	Breast	NR
**Tamim/2008** [12]	NR	1–3, 4–7, 8–13, 14+	Penicillins, tetracyclines, macrolides, cephalosporins, sulfonamides, others	Breast	1–5, 6–10, 11–15
**Tamim/2010** [31]	NR	1–2, 3–5, 6–11, 12+	Penicillins, tetracyclines, macrolides, cephalosporins, sulfonamides, others	Prostate	1–5, 6–10, 11–15
**Tamim/2011** [26]	NR	Q1, Q2, Q3, Q4	Penicillins, tetracyclines, macrolides, cephalosporins, sulfonamides, others	Cervical, ovarian, uterine	1–5, 6–10, 11–15
**Velicer/2004** [27]	NR	1–10, 11–25, 26–50, ≥51 (1–50, 51–100, 101–500, 501–1000, 1000+ days)	Penicillins, tetracyclines, macrolides, cephalosporins, sulfonamides, nitrofurantoins	Breast	NR
**Wang/2014** [33]	NR	Highest vs. second vs. lowest tertile (<7, 7–14, 14+ days)	Beta-lactam, cephalosporins, carbapenems, lincosamides, imidazoles, moxifloxacin	Colorectal	
**Yang/2016** [35]	NR	0–1, 2–4, 5–9, 10–19, 20+	Penicillins, Cephalosporins, Monobactams, Carbapenems, Glycopeptides, Fosfomycin trometamol, Inhibitors of mycobacterial cell wall, Pyrazinamide Combo, Lipopeptide, Aminoglycosides, Tetracyclines, Macrolides, Chloramphenicol. Oxazolidonones, Sulfonamides, Dapsone, Quinolones, Metronidazole, Nitrofurantoins, Ansamycins, Rifabutin, Clofazimine	Liver	<2, 2–5, >5
**Zhang/2008** [30]	NR	1–4, 5–9, ≥10	Penicillins, tetracyclines, macrolides, quinolones, cephalosporins, sulfonamides	Lung	NR

**Legend**: NR, not reported; Q, quartiles; NHL, non-Hodgkin lymphoma; MM, multiple myeloma.

**Table 3:** Main results and subgroup analyses.

Subgroup Analysis	N°	Adjusted OR (95% CI)	*p*	I^2^	*p* for Hetereogeneity	Analysis
***All antibiotic use vs. none***	25	1.18 (1.12–1.24)	<0.001	94%	<0.001	Random
**N° prescriptions: higher vs. none/lower**	21	1.28 (1.14–1.44)	<0.001	96%	<0.001	Random
**Duration of use: higher vs. lower**	6	1.31 (1.11–1.54)	<0.001	95%	<0.001	Random
**Diseases:**						
➢ **Breast**	10	1.15 (1.06–1.24)	<0.001	96%	<0.001	Random
➢ **Colorectal**	5	1.08 (1.007–1.17)	0.03	92%	<0.001	Random
➢ **Gastric**	6	1.06 (1.02–1.1)	0.001	51%	0.06	Fixed
➢ **Esophagus**	4	0.98 (0.93–1.04)	0.6	0%	0.7	Fixed
➢ **Lung**	4	1.29 (1.03–1.61)	0.02	89%	<0.001	Random
➢ **Lymphoma**	4	1.31 (1.13–1.51)	<0.001	90%	<0.001	Random
➢ **Central Nervous System**	2	Not analyzed				
➢ **Pancreatic**	4	1.28 (1.04–1.57)	0.019	89%	<0.001	Random
➢ **Bladder**	3	1.22 (1.08–1.37)	0.001	91%	<0.001	Random
➢ **Renal**	3	1.28 (1.1–1.5)	0.001	89%	<0.001	Random
➢ **Prostate**	6	1.25 (1.1–1.41)	<0.001	97%	<0.001	Random
➢ **Melanoma**	3	1.08 (1–1.17)	0.045	83%	<0.001	Random
➢ **Skin non melanoma**	2	Not analyzed				
➢ **Uterine**	3	0.97 (0.94–1.01)	0.3	4%	0.39	Fixed
➢ **Ovarian**	3	0.95 (0.92–0.99)	0.027	0%	0.86	Fixed
➢ **Cervix**	4	0.75 (0.58–0.96)	0.025	85%	<0.001	Random
➢ **Head and neck**	2	Not analyzed				
➢ **Liver**	4	1.22 (1.05.1.41)	0.008	85%	<0.001	Random
➢ **Biliary tract**	4	1.05 (1.01–1.1)	0.009	20%	0.25	Fixed
➢ **Myeloma**	3	1.36 (1.18–1.56)	<0.001	76%	0.001	Random
➢ **Sarcoma**	1	Not analyzed				
**Type of antibiotics:**						
➢ **Beta-lactams**	16	1.15 (1.12–1.19)	<0.001	89%	<0.001	Random
➢ **Cephalosporins**	14	1.19 (1.13–1.25)	<0.001	81%	<0.001	Random
➢ **Carbapenems**	2	Not analyzed				
➢ **Macrolides**	14	1.11 (1.06–1.16)	<0.001	69%	<0.001	Random
➢ **Tetracyclines**	15	1.06 (1.04–1.09)	<0.001	66%	<0.001	Random
➢ **Quinolones**	10	1.15 (1.09–1.21)	<0.001	80%	<0.001	Random
➢ **Nitrofurantoins**	6	1.05 (0.990–1.1)	0.01	24%	0.28	Random
➢ **Sulfonamides**	14	1.07 (1.03–1.11)	<0.001	74%	<0.001	Fixed
➢ **Aminoglicosydes**	2	Not analyzed				Random
➢ **Nitroimidazoles**	4	1.09 (1.01–1.17)	0.015	54%	<0.001	
➢ **Lincosamides**	2	Not analyzed				Random
**Time elapsed from antibiotic use and incident cancer**	8	1.14 (1.05–1.24)	0.001	89	<0.001	Random
**Type of study:**						
➢ **retrospective cohort**	5	1.16 (1.09–1.23)	<0.001	95%	<0.001	Random
➢ **case-control**	20	1.18 (1.1–1.26)	<0.001	94%	<0.001	Random

**Legend:** OR, odds ratio; N°, number of studies. p = significance; I^2^ = heterogeneity index, vs. = versus.
